# Association between nutrition literacy and cognitive impairment among older adults in Bengbu, China

**DOI:** 10.3389/fnagi.2025.1532279

**Published:** 2025-05-08

**Authors:** Yan Cui, Xiaoting Hu, Xi Tian, Yuhui Sun, Bingyong Zhang, Huaqing Liu

**Affiliations:** School of Public Health, Bengbu Medical University, Bengbu, Anhui, China

**Keywords:** cognitive impairment, nutrition literacy, healthy behavior, China, older adults

## Abstract

**Background:**

Cognitive impairment is a major public health concern. Nutrition literacy (NL) is the capacity of an individual to make informed decisions about nutrition, which is reflected in their eating behaviors and ultimately affects their overall nutritional wellbeing. The association between NL and cognitive impairment is unclear.

**Methods:**

A cross-sectional study was conducted among individuals aged 60 years and above. NL was evaluated via the validated NL-12 scale, and cognitive impairment was identified via a simplified 30-item Chinese Mini-Mental State Examination scale. The association between NL and cognitive impairment was examined via binary logistic regression.

**Results:**

Of the 1,344 study participants, 30.3% had cognitive impairment. Compared with those in the lowest NL quartile, individuals in the highest NL quartile had a lower likelihood of experiencing cognitive impairment [odds ratio (OR) = 0.12, 95% confidence interval (CI) = 0.07–0.20]. This relationship extends to the dimensions of knowledge, understanding, obtaining, interactive, and critical skills. Moreover, the negative association of NL in the Q4 group with cognitive impairment, compared with that in the corresponding Q1 group, was significant regardless of age, sex, exercise status, and socioeconomic status. This association, however, was only evident in older adults who exhibited healthy behavior. Additionally, health behavior significantly moderated the association between NL and cognitive impairment, with an interaction *p* value of 0.018.

**Conclusion:**

Higher levels of NL were associated with lower odds of cognitive impairment in older adults, especially those exhibiting healthier behavior. This study underscores the importance of enhancing NL as a means to mitigate cognitive impairment in older adults. Future research should concentrate on examining interventions that synergize NL with healthy lifestyle practices, ensuring their seamless integration into the daily routines of older adults to address the challenges associated with cognitive impairment effectively.

## 1 Introduction

Cognitive impairment has emerged as a significant risk factor that threatens the health of older individuals, who constitute a considerable portion of the global population. In China, 38.8 million older people are currently affected by cognitive impairment (Jia et al., 2020), and this figure is anticipated to increase to 48.68 million by the year 2060 (GBD 2016 Dementia Collaborators, 2019). More than two-thirds of older people with cognitive impairment are likely to develop dementia within a decade (Hebert et al., 2003). Among ageing people, cognitive impairment is the most common cause of disability. Moreover, they impose an economic burden on patients’ families and society. However, the diagnosis and treatment rates for cognitive impairment are low in China because both awareness and medical experts are lacking. Investigating modifiable factors associated with cognition in this population is essential to prevent cognitive impairment.

Nutrition is a crucial modifiable factor that affects cognitive impairment in older adults (Guarnieri et al., 2024). Epidemiological studies have suggested that increased consumption of fish can mitigate the risk of cognitive impairment (Sasaki et al., 2024) and that increased intake of dietary zinc can improve cognition among older individuals (Meng et al., 2024). However, previous studies have tended to investigate only a single food or nutrient, thus overlooking the intricacy and variety of nutrients. Consequently, the effects of nutrition on cognitive impairment are only partially understood (Sirui et al., 2023; Jia et al., 2024). Changes in one food constituent or nutrient group typically result in compensatory changes in other food constituents or nutrient groups (Zhu et al., 2022). Additionally, evidence shows that the effects of a diet or a combination of nutrients exceed the cumulative effects of the single constituents involved (Jacobs and Orlich, 2014). Adopting an integral nutrition approach could minimize the discovery of coincidental connections, which may otherwise arise when multiple individual components are analysed (Fung and Brown, 2013). Moreover, this approach necessarily accounts for the interactions between multiple nutrients (Jacobs and Orlich, 2014). Hence, the integral nutrition approach can provide a holistic understanding of the relationships between an individual’s nutritional status and the course of their condition.

Nutrition literacy (NL), as an emerging field of study, is derived from the concept of health literacy, where an individual is capable of acquiring, understanding, and applying a wide range of complex nutrition-related information (Krause et al., 2018; Li et al., 2022). NL encompasses both the inherent decision-making ability of the individual (Li et al., 2022) and the external performance of the diet (Taylor et al., 2019). Individuals with high levels of NL are more capable of comprehending the correlation between unhealthy eating habits and disease (Mostafazadeh et al., 2024), thus leading to the adoption of healthier dietary behaviors (Taylor et al., 2019). An improved NL can reduce the burden of disease and alleviate economic and health inequalities (Yarmohammadi et al., 2022). NL is becoming increasingly important in the management, treatment, and prevention of noncommunicable diseases (D’Amato-Kubiet, 2013). In recent years, NL has garnered significant attention due to its crucial role in improving public health, preventing chronic diseases and promoting healthy lifestyles.

However, to date, few studies have explored the association between NL and cognitive impairment. Therefore, this study aimed to analyse the associations among older Chinese adults. The findings of this study can serve as a reference for the implementation of targeted nutrition interventions to enhance cognitive function in older Chinese adults.

## 2 Materials and methods

### 2.1 Study design

This cross-sectional study was conducted from May to July 2023, and a survey on NL and health status was administered to adult residents of Bengbu city, China. Trained investigators conducted in-person interviews via a detailed questionnaire. Every participant provided signed informed consent before data collection. The study was authorized by the Ethics Committee of Bengbu Medical University (approval number 2021099).

### 2.2 Participants

Survey respondents were recruited via stratified, multiple-stage random sampling. The samples were stratified according to urban and rural areas. First, two urban and two rural regions were randomly selected. Subsequently, within these regions, two communities and two villages were randomly selected as sampling units. From these units, 110 households were randomly selected, and adult individuals who met the criteria of consciousness, normal communication ability, and competence in completing the questionnaires were interviewed.

A total of 2,279 individuals participated in the survey, of whom 935 were disqualified for the following reasons: 924 were under the age of 60, 4 provided incomplete information about their NL, and 7 provided incomplete information about their cognitive impairment. Thus, the final sample included 1,344 people aged 60 years or older.

### 2.3 Assessment of NL

NL was evaluated using the validated 12-item NL-12 scale (Mo et al., 2022). This scale comprises two domains, namely, nutrition cognition and nutrition skills, and six dimensions, namely, nutrition knowledge, nutrition understanding, obtaining skills, applying skills, interactive skills, and critical skills. Every question was evaluated via a 5-point Likert scale. The maximum score on the NL-12 scale was 60, with higher scores indicating higher levels of NL. The Cronbach’s alpha for the NL-12 scale in this study was 0.870.

### 2.4 Assessment of cognitive impairment

Cognitive impairment was identified via a simplified 30-item Chinese Mini-Mental State Examination (MMSE) scale, which covers 5 cognitive domains: orientation (0–10 points), immediate recall (0–3 points), delayed recall (0–3 points), attention and calculation (0–5 points), and language and visual space (0–9 points) (Wu et al., 2019b). The education level of the participants was used to determine the threshold scores that defined the presence or absence of cognitive abnormalities. For individuals with primary school education or below, scores below 20 indicate cognitive impairment, whereas for individuals with secondary school education or above, scores below 24 indicate cognitive impairment (Li et al., 2016). In the present study, raw scores on each cognitive domain of the MMSE were converted to age-specific *Z* scores via data from all participants (60–70-year-old group and 71-year-old and older group). Cognitive impairment in each domain of the MMSE was defined as scores that were more than 1.0 standard deviations (SD) below the mean of the same age group (Kondo et al., 2021). These threshold values have been used by several studies on cognitive impairment in older Chinese adults (Wang et al., 2022; Ren et al., 2023). The Cronbach’s alpha for the 30-item Chinese MMSE scale in this study was 0.909.

### 2.5 Covariates

The control variables were sociodemographic characteristics, namely, age (60–70 years or ≥71 years), marital status (married or other), sex, healthy behavior, exercise status (yes or no), socioeconomic status (SES), chronic diseases, and body mass index (BMI). In accordance with previous studies (Liang et al., 2000; Luo and Waite, 2005), SES was evaluated by asking the participants whether they (1) had secondary school education or higher, (2) were born in an urban area, or (3) earned at least 3,000 RMB per month. On the basis of previous studies (Benton and French, 2024), three health-related behaviors (smoking, drinking alcohol and drinking tea) that are commonly used in the Chinese population (Li et al., 2015; Khan and Mukhtar, 2018) were used to assess health behaviors in the older age group. Healthy behavior was evaluated by asking the participants whether they: (1) non-smoking; (2) non-drinking alcohol; and (3) drinking tea. We measured SES and healthy behavior on a scale from 0 to 3. The participants received an additional score of “1” if they answered “yes” to specific questions. Higher scores indicate higher SES or more healthy behaviors. We evaluated chronic diseases by counting the number of participants with diabetes, hypertension, stroke or cerebrovascular disease, heart disease, or lung disease. Chronic diseases were scored as “0” for no history of the condition, “1” for one chronic condition, and “2” for a combination of chronic conditions.

### 2.6 Statistical analysis

NL was categorized into quartiles (Q1–Q4), with Q1 and Q4 indicating the lowest and highest NL levels, respectively. The spread of qualitative variables was evaluated via chi-square analyses. The association between NL and cognitive impairment was evaluated via binary logistic regression. All the models were adjusted for marital status, SES, age, healthy behavior, sex, and chronic diseases. NL was defined in quartiles because this quantitative definition allowed for the *p* value of the linear trend to be calculated. Subgroup analyses were performed for the categories of age, sex, marital status, SES, healthy behavior, and chronic diseases. We also examined the effects of the interaction between these factors and NL on cognitive impairment. The data were analysed via SPSS 24.0 software. Significance was set to *p* < 0.05.

## 3 Results

Table 1 provides the summary statistics. We analysed data from 1,344 participants, of whom 50.1% were 71 years or older in age and 54.5% were women. Among the older adults, 34.0% had one chronic disease, and 37.5% had two or more chronic diseases. Most of the participants were married (78.7%) and exercised (63.7%). The overall SES of the participants was low, with only 17.7% exhibiting the highest SES score. Individuals in the highest quartile of NL were younger, married, did not exercise, and had a higher SES than those in the lowest quartile. Among all the participants, 30.3% (407/1,344) had cognitive impairment, and these individuals tended to be older, did not exercise, and were unmarried women with a lower SES and at least one chronic disease.

**Table 1:** Characteristics of the nutrition literacy study population and prevalence of cognitive impairment.

Variable	*N* (%)	Nutrition literacy *n* (%)				*χ* ^2^	Cognitive impairment *n* (%)	*χ* ^2^
		Q1	Q2	Q3	Q4			
Total	1,344	363 (27.0)	336 (25.0)	323 (24.0)	322 (24.0)		407 (30.3)	
Age						16.357**		23.926***
60–70	671 (49.9)	158 (23.5)	169 (25.2)	155 (23.1)	189 (28.2)		162 (24.1)	
71+	673 (50.1)	205 (30.5)	167 (24.8)	168 (25.0)	133 (19.8)		245 (36.4)	
Sex						17.008**		33.193***
Male	612 (45.5)	139 (22.7)	147 (24.0)	173 (28.3)	153 (25.0)		137 (22.4)	
Female	732 (54.5)	224 (30.6)	189 (25.8)	150 (20.5)	169 (23.1)		270 (36.9)	
Marital status						12.815**		11.511**
Married	1,058 (78.7)	269 (25.4)	255 (24.1)	265 (25.0)	269 (25.4)		297 (28.1)	
Other	286 (21.3)	94 (32.9)	81 (28.3)	58 (20.3)	53 (18.5)		110 (38.5)	
SES						282.603***		145.844***
0	568 (42.7)	242 (42.6)	175 (30.8)	102 (18.0)	49 (8.0)		266 (46.8)	
1	282 (21.2)	70 (24.8)	79 (28.0)	81 (28.7)	241 (43.4)		70 (24.8)	
2	246 (18.5)	35 (14.2)	44 (17.9)	68 (27.6)	99 (40.2)		34 (13.8)	
3	235 (17.7)	13 (5.5)	32 (13.6)	68 (28.9)	122 (51.9)		29 (12.3)	
Healthy behavior						13.41		11.882*
0	169 (12.7)	39 (23.1)	44 (26.0)	52 (30.8)	34 (20.1)		40 (23.7)	
1	321 (24.2)	85 (26.5)	86 (26.8)	76 (23.7)	74 (23.1)		83 (25.9)	
2	677 (50.4)	193 (28.5)	173 (25.6)	152 (22.5)	159 (23.5)		233 (34.4)	
3	162 (12.1)	43 (26.5)	31 (19.1)	37 (22.8)	51 (31.5)		50 (30.9)	
Exercise						79.08***		59.77***
Yes	854 (63.7)	182 (21.3)	190 (22.2)	225 (26.3)	257 (30.1)		196 (23.0)	
No	487 (36.3)	181 (37.2)	145 (29.8)	98 (20.1)	63 (12.9)		210 (43.1)	
BMI						9.359		3.564
Underweight	32 (2.4)	9 (28.1)	8 (25.0)	9 (28.1)	6 (18.8)		12 (37.5)	
Normal	504 (37.8)	140 (27.8)	118 (23.4)	128 (25.4)	118 (23.4)		164 (32.5)	
Overweight	560 (41.9)	143(25.5)	146(26.1)	120(21.4)	151(27.0)		157 (28.0)	
Obesity	239 (17.9)	67(28.0)	61(25.5)	65(27.2)	46 (19.2)		69 (28.9)	
Chronic diseases						19.726**		17.494***
0	383 (28.5)	91 (23.8)	76 (19.8)	114 (29.8)	102 (26.6)		93 (24.3)	
1	457 (34.0)	129 (28.2)	119 (26.0)	94 (20.6)	115 (25.2)		129 (28.2)	
2	503 (37.5)	143 (28.4)	141 (28.0)	115 (22.9)	104 (20.7)		185 (36.8)	

^*^p < 0.05, ^**^p < 0.01, ^***^p < 0.001. SES was calculated as a continuous variable according to the response to education, monthly income (RMB), and place of origin, ranging from 0 to 3, with a higher score indicating a higher SES level. Healthy behavior was calculated as a continuous variable according to the response to non-smoking, non-alcohol, and drinking tea status, ranging from 0 to 3, with a higher score indicating a healthier behavior.

**Figure 1:**
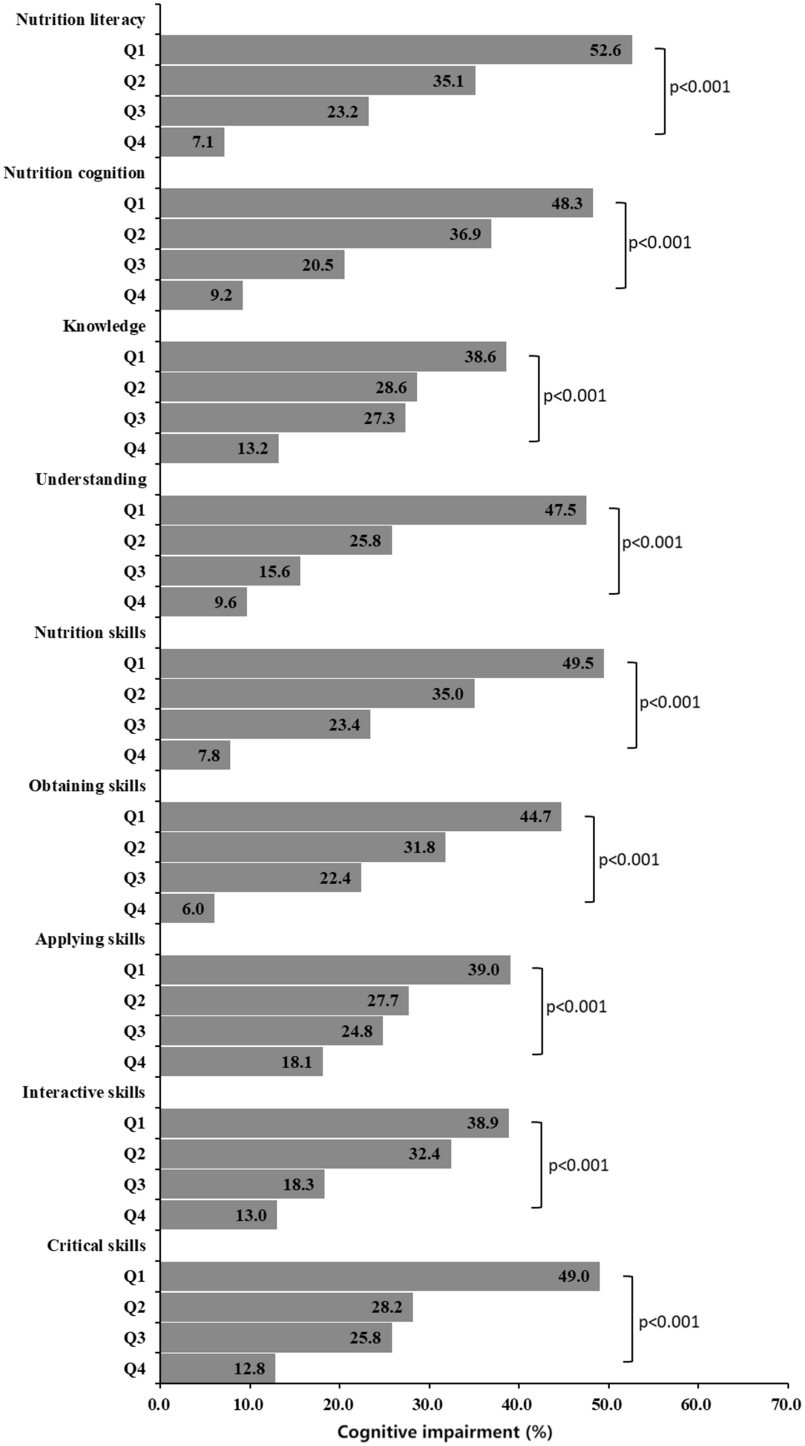
Associations between nutrition literacy and the prevalence of cognitive impairment.

Figure 1 illustrates the prevalence of cognitive impairment according to NL and its different dimensions. Among the individuals with cognitive impairment, 52.6% belonged to the first quartile of NL (lowest level), whereas 7.1% belonged to the fourth quartile of NL (highest level). The difference between them was statistically significant, with a *p* value of less than 0.001. The odds of cognitive impairment decreased with increasing NL. This relationship was observed in both the domains and all six dimensions of NL.

**Figure 2:**
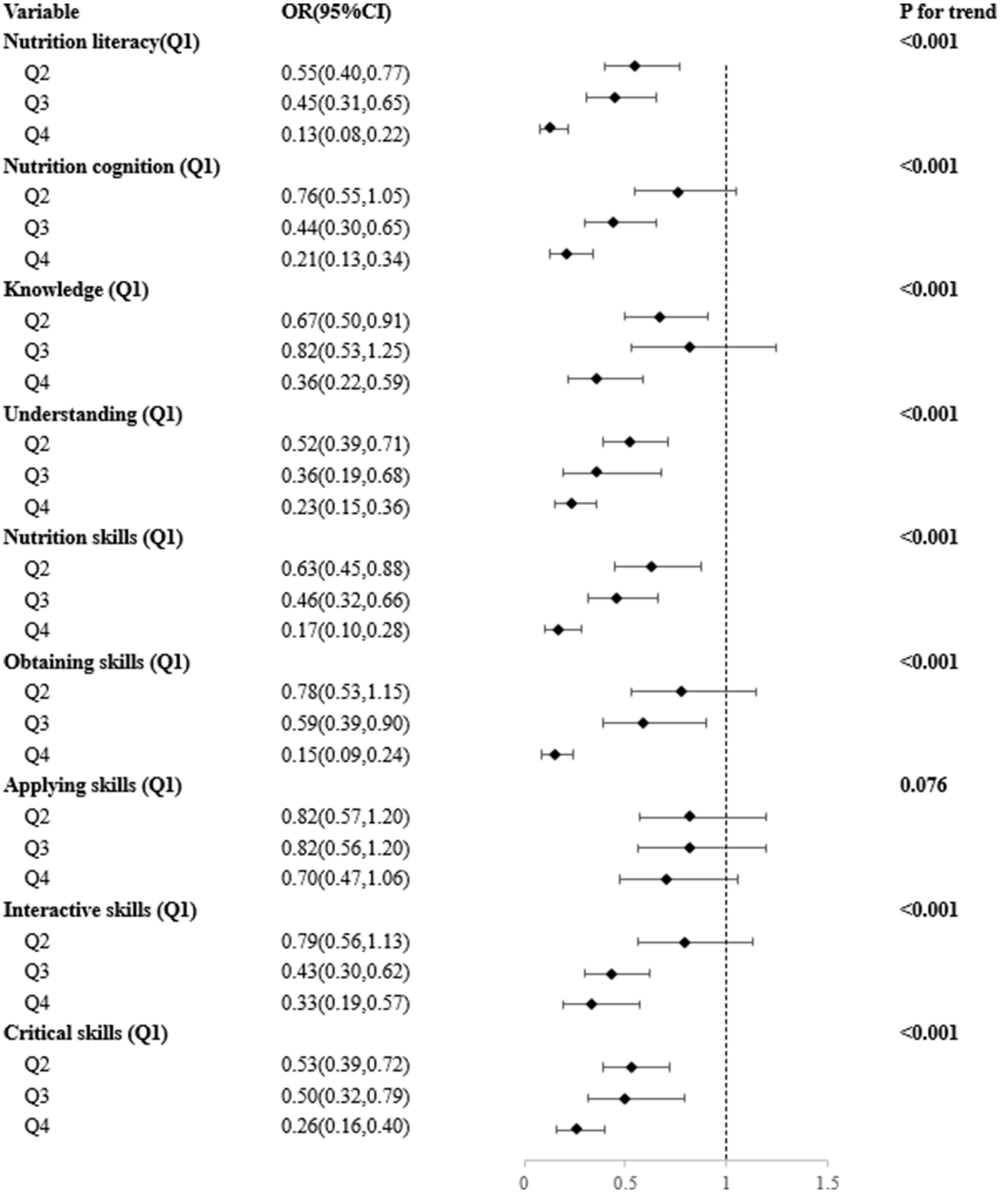
Logistic regression of the association between nutrition literacy and cognitive impairment. All models were adjusted for age, sex, marital status, SES, healthy behavior, exercise status, BMI, and chronic diseases. SES was calculated as a continuous variable according to the response to education, monthly income (RMB), and place of origin, ranging from 0 to 3, with a higher score indicating a higher SES level. Healthy behavior was calculated as a continuous variable according to the response to non-smoking, non-alcohol, and drinking tea status, ranging from 0 to 3, with a higher score indicating a healthier behavior.

Figure 2 shows the results of logistic regression of cognitive impairment according to NL and its different dimensions. After age, sex, healthy behavior, exercise status, BMI, marital status, chronic illnesses, and SES were controlled for, individuals in the highest NL quartile had significantly lower odds of cognitive impairment than those in the lowest NL quartile did [odds ratio (OR) = 0.13, 95% confidence interval (CI) = 0.08–0.22, P for trend<0.001]. This relationship was noted in the domain of nutrition cognition (OR = 0.21, 95%CI = 0.13–0.34, P for trend<0.001), including the dimensions of knowledge (OR = 0.36, 95%CI = 0.22–0.59, P for trend<0.001) and understanding (OR = 0.22, 95%CI = 0.15–0.36, P for trend<0.001), and in the domain of nutrition skills (OR = 0.17, 95%CI = 0.10–0.28, P for trend<0.001), including the dimensions of obtaining skills (OR = 0.15, 95%CI = 0.09–0.24, P for trend<0.001), interactive skills (OR = 0.33, 95%CI = 0.19–0.57, P for trend<0.001), and critical skills (OR = 0.26, 95%CI = 0.16–0.40, P for trend<0.001), but not in applying skills.

Table 2 shows the associations between NL and impaired cognitive domain function. Compared with those in the lowest NL quartile, those in the highest NL quartile had significantly lower odds of impaired orientation (OR = 0.13, 95%CI = 0.05–0.31), immediate recall (OR = 0.12, 95%CI = 0.06–0.26), impaired attention and calculation (OR = 0.20, 95%CI = 0.12–0.33), impaired delayed recall (OR = 0.29, 95%CI = 0.17–0.48), and impaired language and visual space (OR = 0.13, 95%CI = 0.06–0.26).

**Table 2:** Logistic regression of the association between nutrition literacy and impaired cognitive domain.

Impaired cognitive domain	Nutrition literacy (Ref = Q1)		
	Q2	Q3	Q4
Orientation	0.64 (0.43,0.96)*	0.55 (0.33,0.90)*	0.13 (0.05,0.31)***
Immediate recall	0.71 (0.49,1.03)	0.46 (0.29,0.72)**	0.12 (0.06,0.26)***
Attention and calculation	0.56 (0.40,0.79)**	0.54 (0.37,0.79)**	0.20 (0.12,0.33)***
Delayed recall	0.82 (0.58,1.15)	0.63 (0.43,0.92)*	0.29 (0.17,0.48)***
Language and visual space	0.45 (0.31,0.67)***	0.39 (0.24,0.62)***	0.13 (0.06,0.26)***

All models were adjusted for age, sex, marital status, SES, healthy behavior, exercise status, BMI, and chronic diseases. SES was calculated as a continuous variable according to the response to education, monthly income (RMB), and place of origin, ranging from 0 to 3, with a higher score indicating a higher SES level. Healthy behavior was calculated as a continuous variable according to the response to non-smoking, non-alcohol, and drinking tea status, ranging from 0 to 3, with a higher score indicating a healthier behavior.

**Figure 3:**
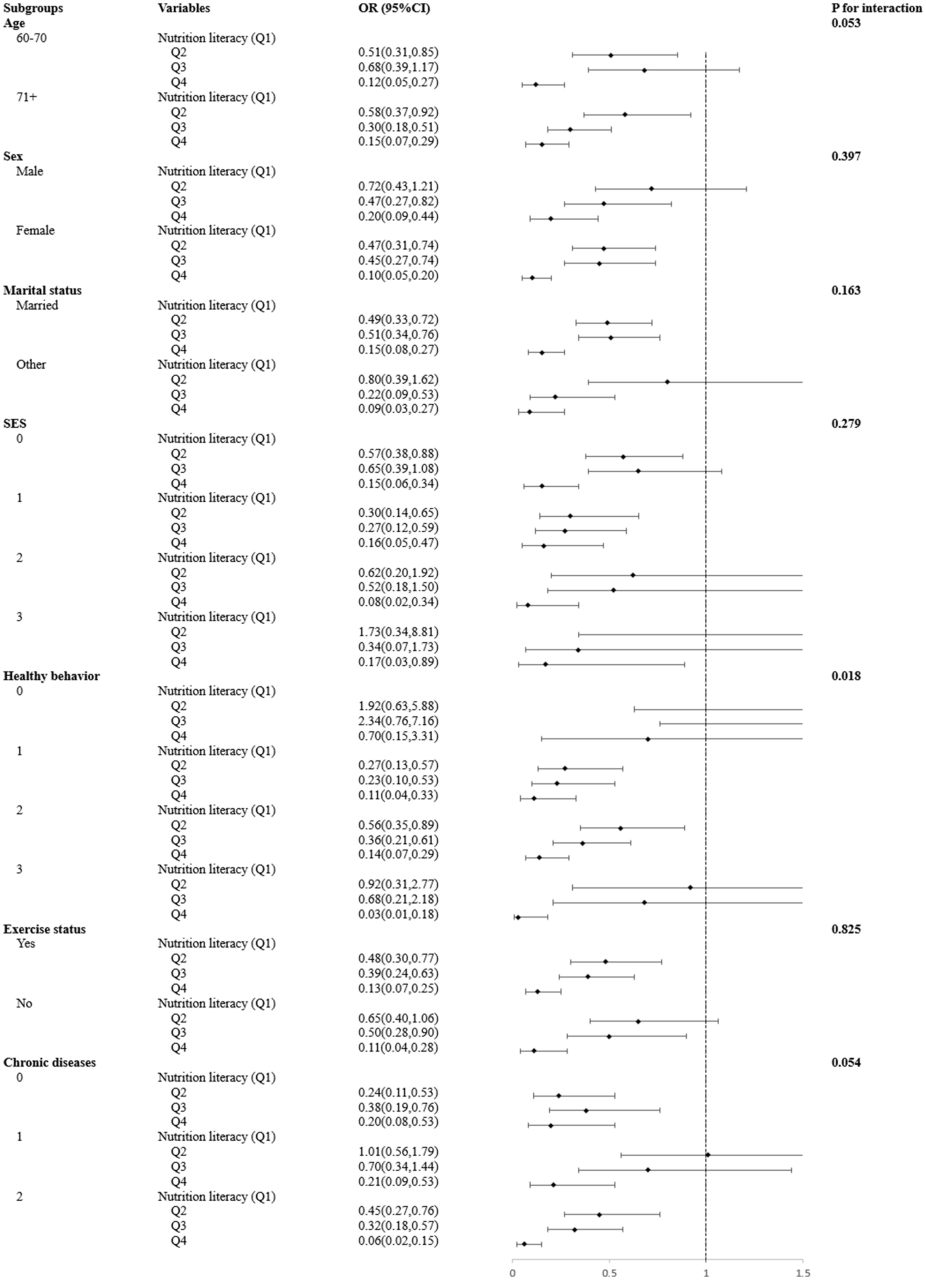
Associations between types of nutrition literacy and cognitive impairment among subgroups and interactions. All models were adjusted for age, sex, marital status, SES, healthy behavior, exercise status, BMI, and chronic diseases. SES was calculated as a continuous variable according to the response to education, monthly income (RMB), and place of origin, ranging from 0 to 3, with a higher score indicating a higher SES level. Healthy behavior was calculated as a continuous variable according to the response to non-smoking, non-alcohol, and drinking tea status, ranging from 0 to 3, with a higher score indicating a healthier behavior.

As shown in Figure 3, stratified analyses revealed a consistent negative effect of the highest NL group (Q4) against cognitive impairment compared with the lowest NL group (Q1) across multiple demographic strata, including age, sex, marital status, exercise status, and socioeconomic status. However, this negative association was only evident in older adults who exhibited healthy behavior (OR = 0.03, 95%CI = 0.01–0.18), but not in those without healthy behavior (OR = 0.70, 95%CI = 0.15–3.31). Further interaction analyses showed that age, sex, marital status, exercise status, and SES did not significantly influence the association between NL and cognitive impairment (all P for interaction>0.05). However, the type of healthy behavior significantly moderated the association between NL and cognitive impairment (P for interaction = 0.018).

## 4 Discussion

This study examined the relationship between NL and cognitive impairment among older adults. The results indicated that higher levels of NL are associated with lower odds of cognitive impairment, particularly in older adults who exhibit healthy behavior. In addition, NL and healthy behavior had an interaction effect on the prevalence of cognitive impairment.

In this study, cognitive impairment affected approximately one-third of all individuals aged 60 years and above in our sample, which is similar to the prevalence of 28.1% reported in Chinese older adults (Qi et al., 2024). This rate was higher than the 7.7% reported by Jiang et al. (2024) for over 20,000 community-dwelling older adults aged 65 years and older in Xiamen but less than the 42.9% reported by Wang et al. (2020) among 1,250 older adults aged 65 years and older in rural areas of northern China. This discrepancy may be attributable to factors such as geographical location, socioeconomic conditions in the studied regions, and specific characteristics of the population. Despite these variations, the findings collectively underscore the severity of cognitive impairment in China’s aging population. Notably, the older, unmarried female population presented a greater risk of cognitive impairment (Alqahtani and Alenazi, 2023; Shin and Cho, 2023). Cognitive impairment is a significant global health problem that imposes a substantial disease burden on society (Aranda et al., 2021). In countries undergoing demographic transition, such as China, the percentage of people aged 60 years and above is growing rapidly (Chen et al., 2022), leading to an increase in the incidence of age-related cognitive impairment and dementia (Salthouse, 2010). Consequently, cognition-enhancing factors must be urgently explored among older members of the population.

Our study revealed a correlation between NL and cognitive impairment in older individuals. Individuals with high levels of NL are better equipped to obtain sufficient nourishment (Doustmohammadian et al., 2020), which is important for maintaining cognitive health (Granot-Hershkovitz et al., 2023). Individuals with higher levels of NL also consume more vegetables (Qi et al., 2023), which can enhance cognitive function in older Chinese adults (Qin et al., 2023). A lack of nutritional knowledge is linked to increased consumption of fried foods (Shen et al., 2021). When any given food is fried, its nutrient content is altered, resulting in the production of acrylamide (Mottram et al., 2002), a hazardous chemical that causes progressive neurodegeneration (Izumi et al., 2022) and can result in cognitive impairment (Pacholko and Iadecola, 2024). Furthermore, the domain of low nutritional skills is significantly linked to a high frequency of consuming takeaway food (Qi et al., 2023). However, takeaway food is low in nutrients and rich in fat (Miura et al., 2009), which can result in cognitive impairment (Morris et al., 2004). In addition, this study did not find an association between application skills and cognitive impairment in older adults. The ability to apply skills refers to the ability to apply nutritional knowledge in real-life situations (D’Amato-Kubiet, 2013). Even in the presence of cognitive decline, older adults may still maintain some nutritional application skills in certain areas. For example, they may follow past eating habits or make simple food choices in a familiar environment. This may reduce the association between the ability to apply skills and cognitive impairment in older people. Notably, NL is associated with all cognitive domains, including orientation, immediate recall, attention and calculation, delayed recall and language and visual space. Therefore, maintaining a high level of NL is essential for preventing cognitive impairment in older individuals.

According to the results of the subgroup and interaction analyses, healthy behavior significantly modified the relationship between NL and cognitive impairment. The association between NL and cognitive impairment was more pronounced among individuals who displayed healthy behavior. Healthy behavior is known to be strongly associated with cognitive impairment (Jin et al., 2021). The adoption of improved healthy behavior can potentially reduce the risk of cognitive deterioration (Erickson et al., 2022). Tobacco and alcohol consumption, recognized as unhealthy behaviors, are two major public health problems worldwide, including in China (Chang et al., 2022). They are risk factors for cognitive impairment (Sanna et al., 2023; Hong et al., 2024). Strengthening tobacco and alcohol control can reduce cognitive decline (Wu et al., 2019a). Tea consumption, as a healthy behavior, is common in the daily life of Chinese people, and long-term tea consumption can help improve cognition in older adults (Milani et al., 2024). Additionally, higher levels of healthy behavior are closely linked to a nutritious diet (Liao et al., 2019), which suggests that promoting healthy behavior can strengthen the association between NL and cognitive impairment. Thus, targeted NL interventions should be accompanied by the promotion of health lifestyles, and older people will benefit from joint actions in public health practice settings.

Our study also revealed that the effect of NL on the incidence of cognitive impairment was more pronounced in older adults than in the oldest adults in the population. The influence of NL on cognitive impairment may decrease with age, likely because cognitive impairment becomes more common as people age (Salthouse, 2010). Therefore, younger age is associated with better cognitive function among older adults. Higher levels of NL are more common in older adults with a younger age (Sanlier et al., 2024), which enables them to maintain better healthy-eating behavior (Liao et al., 2019) and, consequently, to improve cognition (van Soest et al., 2024). A study revealed that sex, age, exercise status and NL were independently associated with cognitive impairment. Cognitive decline is gradual with age (Salthouse, 2010), but older adults with high levels of NL are still able to improve cognitive function through healthy dietary choices (Qi et al., 2023; Qin et al., 2023), suggesting that nutrition literacy can partially compensate for age-related cognitive decline, creating a generalized protective effect across ages. In addition, although women are at greater risk of cognitive impairment than men are at risk (Shin and Cho, 2023), the total NL of men and women are not significantly different from each other (Li et al., 2022). Exercise reduces the risk of cognitive impairment in older adults, but in the case of physically inactive older adults, optimizing their diet may still indirectly reduce the risk of cognitive impairment (Doustmohammadian et al., 2020; Granot-Hershkovitz et al., 2023), thus somewhat diminishing the impact of exercise factors. In addition, previous studies have shown that high levels of NL are strongly associated with high SES (Camargo et al., 2022; Singh et al., 2024), but the interaction between individual SES and cognitive impairment is controversial (Walhovd et al., 2022; Wang et al., 2023). A recent systematic evaluation demonstrated that SES is positively associated with level of cognition (Wang et al., 2023). However, another cohort study by Walhovd et al. (2022) reported that SES is not consistently correlated with brain function and cognition. Our findings that the link between NL and cognitive impairment did not vary according to SES corroborate the results of Walhovd et al. (2022). These results are illuminating insofar as they imply that, among individuals of diverse sexes and SES, a higher level of NL is still linked to a reduced likelihood of cognitive impairment. Therefore, physicians should recommend that older adults increase their levels of NL to improve cognition, regardless of their age, sex, exercise status and SES. Additionally, longitudinal studies or intervention trials among the older population are necessary to validate the protective effect of increased NL on cognitive impairment.

The strength of this study is that it is the first to investigate the relationship between NL and cognitive impairment among older adults. This study also has several limitations. First, the data were obtained via a cross-sectional approach, which enabled the examination of the relationship between NL and cognitive impairment but precluded the inference of causal relationships between them. Second, although the MMSE has been validated in population studies, it is not a clinical diagnostic tool for cognitive impairment. Third, the sample was limited to one city in Anhui Province. Therefore, caution should be exercised when extrapolating the results to other ethnic groups. Fourth, energy intake, which may interact with the association between NL and cognitive impairment, was not considered in this study. Additionally, any potential effects of drug treatments for chronic diseases on the interpretation of results were not considered. Future studies should prioritize longitudinal designs to more precisely determine causal relationships, expand population samples, and systematically consider factors such as energy intake and drug treatments.

## 5 Conclusion

In conclusion, higher levels of NL were associated with a lower prevalence of cognitive impairment. This association was strengthened in older adults who exhibited healthy behavior. Increasing the NL may be beneficial for improving cognitive impairment in older adults, especially those who exhibit healthier behavior. Our findings stress that intervention measures or programs that simultaneously target NL and healthy lifestyles should be recommended to public workers in the community to promote the health of older people. Cohort studies or intervention trials are necessary to further validate the relationship between NL and cognitive impairment.

## Data Availability

The raw data supporting the conclusions of this article will be made available by the authors, without undue reservation.
